# A gluten-free diet does not alter performance outcomes in nonceliac athletes undergoing sprint interval training: a pilot trial

**DOI:** 10.3389/fspor.2025.1646563

**Published:** 2025-10-23

**Authors:** Denise Zdzieblik, Anna Zierke, Tobias Waldvogel, Albert Gollhofer, Daniel König

**Affiliations:** ^1^ Department for Nutrition, Institute for Sports and Sports Science, University of Freiburg, Freiburg, Germany; ^2^ Centre for Sports Science and University Sports, Department for Nutrition, Exercise and Health, University of Vienna, Vienna, Austria; ^3^ Faculty of Life Sciences, Department for Nutrition, Exercise and Health, University of Vienna, Vienna, Austria

**Keywords:** gluten-free diet, endurance, performance, respiratory exchange ratio, fat oxidation, ramp incremental test, sprint interval training

## Abstract

**Introduction:**

The popularity of gluten-free diets (GFD) among athletes has increased due to perceived benefits for performance, well-being, and body composition, despite limited evidence in non-celiac individuals.

**Methods:**

This parallel-group pilot study evaluated the effects of a 6-week GFD vs. a mixed diet (MD), both combined with sprint interval training (SIT), on metabolic and performance-related parameters in 15 male endurance athletes (GFD: *n* = 6; MD: *n* = 9). Outcomes included body composition, time trial performance (distance during a 60 min run on a 400-metre track), metabolic (respiratory exchange ratio (PER), substrate oxidation rates, maximal fat oxidation [MFO], and FatMax—intensity at which MFO occurs), and performance-related (ventilatory threshold [VT], respiratory compensation point [RCP], peak oxygen uptake [V̇O_2_peak], and time to exhaustion [TTE]) markers during a ramp incremental running test. Gastrointestinal quality of life (GIQLI) was also assessed.

**Results:**

The GFD group achieved significant reductions in weight and BMI compared to the MD group (Δweight: GFD: −2.70 [−3.20 to −1.73] kg vs. MD: −0.30 [−1.75 to 1.35] kg, *p* = 0.018, *r* = 0.569; ΔBMI: GFD: −0.75 [−1.00 to −0.50] kg·m^−^² vs. MD: −0.10 [−0.55 to 0.35] kg·m^−^², *p* = 0.026, *r* = 0.549). Both groups demonstrated improved time trial distance (Δ: GFD: 0.76 [0.56 to 1.57] km vs. MD: 0.60 [0.50 to 0.90] km, *p* = 0.313, *r* = 0.328) and TTE (Δ: GFD: 1.24 [0.61 to 1.80] min vs. MD: 0.70 [0.19 to 0.92] min, *p* = 0.088, *r* = 0.442), with V̇O_2_peak increases appearing more pronounced in the GFD group (ΔV̇O₂peak: GFD: 9.10 [1.80 to 12.38] ml·kg^−1^·min^−1^ vs. MD: 3.20 [−1.95 to 10.40] ml·kg^−1^·min^−1^, *p* = 0.388, *r* = 0.246). Group differences in metabolic changes were mixed (ΔMFO: GFD: 0.075 [−0.070 to 0.190] g·min^−1^ vs. MD: 0.200 [−0.145 to 0.310] g·min^−1^, *p* = 0.607, *r* = 0.152; ΔFatMax: GFD: −12.90 [−23.56 to 1.79] %V̇O₂peak vs. MD: 1.00 [−10.23 to 12.11] %V̇O₂peak, *p* = 0.181, *r* = 0.365), and GIQLI scores showed no significant changes.

**Conclusion:**

Although a GFD showed modest benefits for weight management and aerobic performance, its metabolic effects were variable, and challenges with nutritional deficiencies highlight the need for caution in non-celiac athletes.

## 1 Introduction

In genetically predisposed individuals, prolamins can cause intolerance reactions collectively referred to as gluten-related disorders. In celiac disease, prolamins trigger an inflammatory response primarily in the upper small intestine. In the case of wheat allergy, an allergic reaction to specific components of wheat occurs, while other types of grain are generally tolerated. While a gluten-free diet (GFD) is essential for celiac disease, its necessity for healthy non-celiac individuals remains debated (1).

The popularity of a GFD is increasing among competitive athletes due to its perceived benefits on well-being, training adaptation, and body weight management (2). In an online survey conducted by Lis et al. (3) with 910 non-celiac athletes (58% female, from recreational to Olympic level), 41% reported following a gluten-free or reduced-gluten diet more than half of the time, predominantly endurance athletes (70%). Only 13% followed the diet for a diagnosed medical condition, while 57% self-diagnosed gluten sensitivity, often citing gastrointestinal symptoms (16.7%) alone or combined with others such as fatigue. Overall, 84% reported symptom improvement after removing gluten. While only 5%–10% of the general population clinically benefit from a GFD, a substantially higher proportion of non-celiac athletes report adopting the diet, often due to perceived gastrointestinal or other symptoms associated with gluten (3).

At the same time, approximately 20%–50% of endurance athletes experience exercise-induced gastrointestinal symptoms, which are attributed to increased intestinal permeability, altered transit times, reduced intestinal blood flow, and impaired absorption of carbohydrate, water, and electrolytes during physical activity (4). Dietary factors, including fiber content, the type and quantity of carbohydrates, and specific wheat components in sensitive individuals, can also contribute to gastrointestinal symptoms (5).

While a GFD is increasingly adopted by athletes, its overall impact on health and performance in non-celiac individuals remains uncertain. Avoiding gluten-containing foods can reduce total carbohydrate intake, including key sources such as whole grains. This is particularly relevant for endurance exercise lasting longer than 75–90 min, where carbohydrate serves as the primary energy source (6, 7). Nevertheless, these limitations can often be addressed through appropriate dietary planning and the use of alternative carbohydrate sources (8, 9). At the same time, the exclusion of whole grains may reduce the intake of essential micronutrients, including magnesium, calcium, iron, vitamin B12, vitamin D, and folic acid (10). Although such risks are plausible, some studies assessing dietary intake in celiac patients adhering to a long-term GFD have not found a significant risk of inadequate vitamin and trace element intake, based on food records, while clinical nutrient status was not directly assessed (11). Thus, while potential nutritional concerns exist, they are not necessarily inevitable and depend largely on the individual dietary context.

Nevertheless, the existing body of evidence does not yet provide sufficient support for the notion that a GFD offers clear benefits in terms of performance enhancement or gastrointestinal symptom management for non-celiac athletes (3, 8, 12, 13). To date, only one clinical study has investigated the effects of a 7-day GFD on performance-related, inflammatory, and gastrointestinal parameters in non-celiac athletes (14). The results of that study indicated that a short-term GFD had no significant impact on endurance performance, gastrointestinal symptoms, well-being, or inflammation markers in non-celiac cyclists.

Given the limited evidence from short-term effects of a GFD in non-celiac athletes, the present investigation was conceived as an initial pilot trial to compare the effects of a 6-week GFD and a mixed diet (MD), both combined with sprint interval training (SIT), on metabolic and performance-related parameters in healthy male endurance athletes. A small-scale, matched-group design was, therefore, used to generate preliminary evidence and effect size estimates, while also evaluating key practical aspects such as recruitment, adherence, and data completeness. This study thus aims to contribute to a better understanding of the effects of diet on endurance performance and metabolic parameters in non-celiac athletes, and to inform the design of a subsequent, adequately powered trial.

## 2 Material and methods

### 2.1 Study design and participants

The study was conducted as a monocentric, prospective, open-label, matched-group pilot trial at the University of Freiburg, Germany. The primary objective was to assess the additional effects of a GFD on metabolic and performance-related parameters, beyond the effects of a standardized SIT regimen. Moreover, feasibility objectives were evaluated, including recruitment and retention rates, adherence to dietary and exercise protocols, and completeness of outcome data, to inform the design of a future, adequately powered trial. Although the CONSORT 2010 extension is primarily aimed at randomized pilot and feasibility trials, several of its key principles (e.g., clear feasibility objectives, reporting of recruitment and adherence, preliminary effect size estimation) were applied to this non-randomized, matched-group pilot trial (15). Supervised SIT sessions took place at the University of Freiburg between 8 am and 8 pm, with participants assigned to either a GFD or a MD, both provided *ad libitum*.

A total of 20 healthy male, non-professional endurance athletes, aged 18 to 50 years, were recruited. Participants were classified as trained/developmental athletes, engaging in sport-specific endurance training 2 to 5 times per week, identifying with an endurance sport, and intending to compete at local-level events without national or international representation (16). The groups consisted of 10 athletes each, with one group following the GFD (GFD-G) and the other following the MD (MD-G). Due to the limited sample size and to minimize baseline variability in performance-related parameters, participants were matched based on their peak oxygen uptake (V̇O₂peak) values. They were ranked according to their V̇O₂peak and then alternately assigned to either the GFD-G or MD-G in a pairwise manner, ensuring comparable aerobic fitness levels between groups at baseline. The supervising investigator was responsible for assigning participants to their respective groups.

The sample size for this exploratory pilot trial was pragmatically determined based on a prior study investigating the effects of a short-term GFD on athletic performance in non-celiac athletes (14) and on methodological recommendations for pilot studies suggesting approximately 10 participants per group (17). As this was a pilot study, the sample size was chosen to assess feasibility and to provide preliminary variability estimates across the selected outcome measures. No single primary outcome was pre-specified for sample size calculation. For illustration, based on the variability in the measured outcomes, a future main trial aiming to detect a moderate effect size (Cohen's d = 0.5) with α = 0.05% and 80% power would require approximately 64 participants per group. This calculation is based on a two-tailed independent samples t-test using G*Power 3.1 (University of Düsseldorf, Germany). Exclusion criteria included a prior diagnosis of celiac disease, health problems during or after physical activity, unstable weight or eating behaviors, adherence to special diets (e.g., vegan diet), and contraindications to physical activity (e.g., cardiovascular, metabolic, or renal diseases), as defined by the American College of Sports Medicine guidelines (18).

The study protocol received approval from the Ethics Committee of the University of Freiburg (ETK: 208-18). This study was part of a registered trial (DRKS00025708) comprising two independent sub-studies investigating the effects of different dietary interventions in combination with SIT on metabolic and performance-related parameters in healthy male athletes. For context, one sub-study previously reported on a paleolithic diet (19), the present investigation focuses on a GFD, using the same MD-G as control and identical training protocol to ensure methodological consistency across both sub-studies. Given the exploratory nature, limited sample sizes, and testing feasibility, this approach allowed multiple interventions to be compared without separate control groups (20–22). Reuse of data from the previous sub-study is acknowledged under a CC BY-NC 4.0 license. Although both interventions exclude gluten, they differ conceptually: the paleolithic diet represents a broad evolutionary nutrition approach excluding multiple food groups, whereas the GFD specifically targets gluten avoidance (10, 23, 24). The current dietary intervention specifically assesses whether gluten exclusion alone has measurable effects in non-celiac athletes.

**Figure 1:**
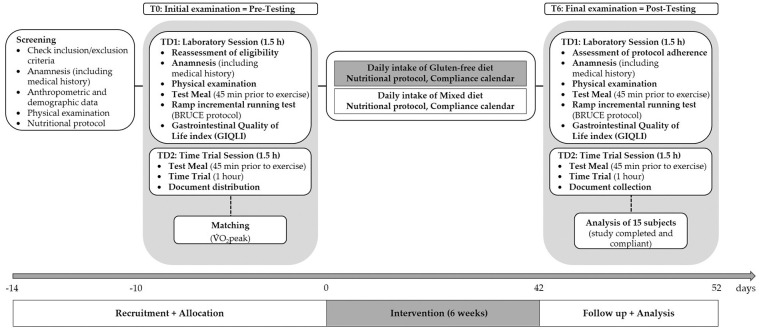
Overview of the study schedule, including baseline (T0) and follow-up (T6) assessments, and the six-week intervention. TD, testing day.

The study spanned approximately nine weeks, including participant screening, baseline (T0) and follow-up (T6) assessments, and the six-week intervention. All participants began the intervention simultaneously. Baseline and follow-up assessments were conducted using multiple morning time slots to accommodate all participants. Each laboratory session lasted around 1.5 h per participant; overlapping slots were possible, as only the treadmill segment required exclusive use. Time trial sessions were scheduled on separate days, with a minimum of two days between sessions to ensure sufficient recovery. The same spacing rules applied between time trial testing and training sessions. Post-testing (T6) followed the same procedure immediately after the intervention. An overview of the study phases and timeline is provided in Figure 1.

After providing written informed consent, participants underwent a screening process that included a detailed medical history questionnaire to confirm eligibility, ensure the inclusion criteria were met, and rule out any risk factors that could be exacerbated by the exercise protocols. During this session, anthropometric measurements (including height, weight, age) were obtained using a calibrated stadiometer and digital BIA scale (OMRON BF-500 Medizintechnik GmbH, Mannheim, Germany), respectively, with participants wearing light clothing and no shoes. Blood pressure was measured on the participant's dominant arm using a validated automated oscillometric device (OMRON M6 Comfort, OMRON Healthcare, Kyoto, Japan). Measurements followed standardized recommendations, with participants seated comfortably, arm supported at heart level, and after a minimum 5 min rest in a quiet environment to ensure accuracy (25).

Body composition assessments were conducted at baseline (T0) and after the intervention (T6), following the procedures described in the efficacy outcomes. Endurance capacity was evaluated using a ramp incremental test and a one-hour time trial at both T0 and T6. Given that substrate oxidation during exercise, as reflected by the respiratory exchange ratio (RER), is influenced by pre-exercise nutrition (26), participants consumed two bananas (125 g each, providing a total of 50 g carbohydrate) 45 min before these tests (27). All study visits, including screening, T0, and T6, were scheduled for the same time in the morning, with participants required to fast for 12 h beforehand. Additionally, they were instructed to empty their bladders before assessments. To ensure proper hydration, participants consumed 1 liter of water the evening prior and 0.5 liters on the morning of each visit (28). Alcohol intake was prohibited for 48 h before any evaluation.

### 2.2 Efficacy outcomes

The participants' body composition (fat free mass and fat mass) was assessed by using a bioelectric impedance analysis (BIA) with a high reliability for evaluating body composition in physically active adults (29, 30). Participants were assessed on the BIA scale (OMRON BF-500 Medizintechnik GmbH, Mannheim, Germany). The typical measurement variability (coefficient of variation) for this BIA device series is approximately 1.3% for fat mass indicating that changes exceeding these thresholds can be considered true changes rather than measurement error (31). According to the manufacturer's recommendation, participants were measured in the morning at the same daytime following a fasted period and under the same environmental conditions at each study visit. In addition, the systolic and diastolic blood pressure were recorded at rest as described above.

Spiroergometry (Geratherm Respiratory GmbH, Germany) was conducted on a treadmill (RAM Model 770CE, United Kingdom) in accordance with evidence from recent studies indicating that SIT significantly influences V̇O_2_max/peak (32, 33). To assess respiratory parameters (RER, V̇O_2_peak, ventilatory threshold [VT], and respiratory compensation point [RCP]), the BRUCE ramp protocol [as illustrated in Supplementary Figure S1 of a previously published investigation by Zdzieblik et al. (19)] was employed. During the initial phase of the protocol (up to 14 min and 20 s), the treadmill grade was progressively increased by 0.2%–0.4% every 20 s. After this, the incline adjustment was set to 0.5% increments every 40 s. The treadmill speed began at 2.7 km/h (1.7 mph) and was raised by 0.2 km/h (0.1 mph) every 20 s from 2 min and 40 s until the 14 min mark. Subsequently speed increased every 40 s (34). The ramp test was preceded by a 3 min rest phase followed by a warm-up period, which included 3 min of light constant-load exercise at 1.7 mph. This preparatory phase aimed to ensure adequate tissue saturation with carbon dioxide, thereby minimizing the risk of misinterpreting the VT (35). VT is determined during incremental exercise as the point where ventilation (VE) begins to increase disproportionately relative to oxygen uptake (V̇O_2_), signaling the transition from aerobic to anaerobic metabolism (36). RCP is marked by a pronounced rise in VE relative to carbon dioxide production (V̇CO₂). Both VT and RCP were identified graphically using the V-slope method, which involves plotting V̇CO₂ against V̇O₂ and VE against V̇CO₂ to detect the respective transition points. These thresholds were evaluated independently by three blinded reviewers to ensure accuracy (36). In addition, V̇O_2_peak was calculated from the highest 30 s V̇O_2_ average during the ramp test (37), and time to exhaustion (TTE) was recorded. Substrate oxidation was also assessed, with maximal fat oxidation (MFO) defined as the highest measured fat oxidation rate, and FatMax as the exercise intensity (%V̇O₂peak) at which MFO occurred (38).

Carbohydrate and fat oxidation rates were calculated using the following Equations (1) and (2) assuming a negligible protein oxidation (39, 40).

(1) $$\mathrm{Carbohydrate}\;\mathrm{Oxidation}\;(g\cdot \mathrm{mi}n^{-1})=(4.585\times V\text{·}CO_{2})\;-\;(3.226\times V\text{·}O_{2})$$

(2) $$\mathrm{Fat}\;\mathrm{Oxidation}\;({g\cdot \min}^{-1})=(1.695\times V\text{·}O_{2})\;-(1.701\times V\text{·}CO_{2})$$

To assess the RER and substrate oxidation rates, the area under the curve (AUC) was calculated using the trapezoidal method, from the start of the test until the final minute completed by all participants before exhaustion. Substrate oxidation rates were determined only for exercise intensities below the VT, as substrate oxidation estimates become unreliable at higher intensities due to bicarbonate buffering and non-metabolic CO₂ production (26). The inclusion of RER and substrate oxidation rates as outcomes was based on their sensitivity to diet-related changes and their relevance for evaluating substrate utilization during exercise (26). For the prolonged endurance performance assessment, participants completed a fixed-duration running time trial outdoors on a 400 m tartan track under comparable environmental conditions (same location, dry surface). Ambient temperature and humidity were monitored and documented during each session (overall range across all tests:16–26°C, 60%–80% relative humidity). Following a 5 min warm-up on a 400-meter track, the distance covered [km] during the 60 min trial was recorded using a GPS device (Polar M200, Kempele, Finland) (41). Quality of life was measured using the Gastrointestinal Quality of Life Index (GIQLI), a 36-item questionnaire covering five domains: gastrointestinal symptoms (19 items, max 76 points), physical function (7 items, max 28 points), emotional well-being (5 items, max 20 points), social dimension (4 items, max 16 points), and therapeutic component (1 item, max 4 points). The total score ranges from 0 to 144, with higher values reflecting better quality of life. A score below 112 indicates reduced quality of life (42, 43). For gluten sensitivity, the gastrointestinal domain is most relevant, while physical and emotional domains also reflect common symptoms such as fatigue and reduced well-being (44–46).

The statistician responsible for the analysis remained blinded during all procedures. Data unblinding occurred only after data collection was complete and the database was locked, following standard data management protocols.

### 2.3 Nutritional guidelines

After attending an information evening to get familiar with nutritional concepts, participants were instructed to follow the dietary pattern according to their respective group (GFD-G or MD-G) over the time course of 6 weeks. The diet of the GFD-G included fresh fruits, vegetables, unprocessed meats, fish, gluten-free grains (e.g., rice, quinoa, corn, buckwheat), legumes, nuts, and dairy products, unless flavored or processed with gluten. Gluten-free flours (e.g., rice, almond, coconut) and certified gluten-free processed foods (e.g., bread, pasta) were permitted.

Excluded from the diet were all wheat-based products (e.g., bread, pasta), barley, rye, oats (unless certified gluten-free), and processed foods containing gluten additives (e.g., malt, wheat starch). Alcoholic beverages such as beer and most processed foods containing gluten-based ingredients (e.g., modified starches) were also restricted. To prevent cross-contamination, careful attention was given to food handling and preparation. To assist participants in identifying gluten-free products, foods labelled with the “Gluten-Free” symbol, often accompanied by a crossed-out wheat ear symbol, were included. This symbol is a licensed trademark of the German Celiac Society (“Deutsche Zöliakie Gesellschaft”). Participants of the MD-G were instructed not to change their nutritional habits. In addition, participants of both groups were asked to complete a three-day nutrition protocol, using digital three-day food diaries provided via the Nutriguide online platform (Nutri-Science GmbH, Pohlheim, Germany), covering two weekdays and one day at the weekend, before, after three and after six weeks of intervention. Participants received a personalized link for each recording period, through which they could either select foods from the integrated German Nutrient Database (Bundeslebensmittelschlüssel, BLS) or enter additional items via a free-text option. They were instructed to record all foods, beverages, and dietary supplements consumed. Portion sizes, meal times, preparation methods, and product brands were noted where possible. Quantification of portion sizes was primarily based on grams or milliliters, but household measures such as tablespoons, teaspoons, and cups were also accepted. All entries were completed by participants themselves and, once submitted, were reviewed by a qualified nutritionist for completeness, plausibility, and consistency with the study protocol. Where entries were ambiguous, incomplete, or implausible, the nutritionist contacted participants directly for clarification or correction. Verified records were then analyzed using the Nutriguide software (Nutri-Science GmbH, Pohlheim, Germany) by the same investigator to maintain consistency in coding and food selection. To ensure quality control, questionable entries were flagged and discussed within the study team before final confirmation. Energy and nutrient intakes were screened for potential under-reporting using plausibility checks (e.g., unusually low total energy intake relative to reported activity levels). While no datasets were excluded on this basis, all flagged cases were clarified with participants whenever possible, and this potential source of error is acknowledged in the study limitations.

### 2.4 Exercise protocol

Participants were instructed to maintain their usual physical activity levels, excluding the supervised short-term interval training (SIT). The SIT protocol utilized the Wingate anaerobic test format as the exercise component, specifically designed for an active population accustomed to endurance training (47, 48). Each supervised session began with a five-minute warm-up, performed at 70%–80% of maximal heart rate, below the VT (49). The main training consisted of 30-second all-out sprints, followed by a 4 min recovery phase at the same intensity as the warm-up. The number of repetitions was progressively increased: weeks 1–2 involved four repetitions, weeks 3–4 included five, and weeks 5–6 involved six repetitions. A cool-down was performed under the same conditions as the warm-up and recovery phases. All training sessions were conducted at the University of Freiburg between 8 am and 8 pm, with a total of 18 sessions completed during the 6-week intervention.

### 2.5 Statistical analysis

All data are presented as median and interquartile range (IQR) unless stated otherwise. SPSS statistics (IBM SPSS Statistics for Windows, Version 25.0. Armonk, NY: IBM Corp.) was used for all statistical analyses. In the present trial, endpoints had been defined with no hierarchy. The statistical evaluation was performed to determine an adequate sample size and the primary outcome of a main RCT study, which will be designed on the basis of the present study protocol. All the tests in the descriptive analysis were performed as two-sided tests and the significance level was set at α = 0.05.

Due to the small sample size and uneven number of subjects per group, the homogeneity of the baseline values between the study groups was checked using the Mann–Whitney *U*-test. In addition, the mean differences obtained from both groups were compared using the Mann–Whitney *U*-test. The significance of changes in the respective endpoints during the intervention period within groups were analyzed with the Wilcoxon signed-rank test. For nutrients with an Average Requirement (AR), the proportion of participants below the AR is reported, based on the European Food Safety Authority (EFSA) in their Scientific Opinion on Dietary Reference Values for nutrients. For nutrients with only an Adequate Intake (AI), intakes are described relative to the AI without inferring inadequacy (50). To assess dietary intake during the intervention, data from weeks 3 and 6 were averaged and compared with baseline values.

The magnitude of change in the respective outcomes was expressed as the rank-based effect size r, calculated from the *Z*-value of Mann–Whitney or Wilcoxon tests, for differences between groups at the end of the investigation and within groups between baseline and post-intervention, using the following Equation (3):

(3) $$\frac{Z}{\sqrt{N}}$$

where N is the total number of observations. Effect sizes were interpreted according to conventional thresholds: small effect: 0.10 ≤ r < 0.30; medium effect: 0.30 ≤ r < 0.50; large effect: r ≥ 0.50 (51).

## 3 Results

### 3.1 Feasibility outcomes and subject characteristics

**Figure 2:**
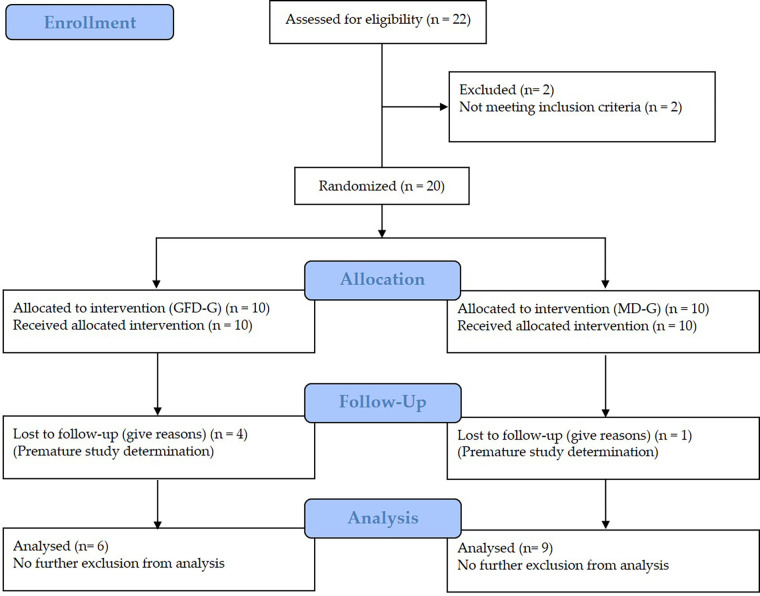
Flow chart of subject recruitment, randomization, and follow up.

Recruitment took place over four weeks, during which 35 athletes initially expressed interest; however, many withdrew prior to the formal screening (*n* = 11) or were excluded based on a preliminary telephone screening (*n* = 2). Ultimately, 22 athletes were screened. A total of 20 men (91%) met the inclusion criteria and were enrolled in the study (Figure 2). Of these, 15 participants completed the trial and were subsequently included in the final analysis, resulting in a retention rate of 60% in the GFD-G (6/10) and 90% in the MD-G (9/10). Participant dropout was attributed to voluntary withdrawal, as individuals opted not to continue with the dietary intervention or training program. No adverse events or pathological findings related to the intervention were identified through routine anamnesis or the compliance calendar. The analysis of the training protocols revealed no significant differences (*p* = 0.529) between the GFD-G (17 ± 1) and MD-G (17 ± 1) in terms of the number of completed training sessions. Dietary adherence, based on self-reported food records and compliance calendars, was estimated at 100% in both the GFD-G and MD-G. Completeness of key outcome data was 100%, with no major protocol deviations.

At baseline, no significant differences between groups were observed in the outcome parameters reported in the following sections. Age was comparable between groups (GFD-G: median 24.5 years, IQR 22.5–31.0; MD-G: median 26.0 years, IQR 22.0–28.0; *p* = 0.955), whereas body height was significantly greater in the GFD-G (1.85 m, IQR 1.82–1.87) compared to the MD-G (1.79 m, IQR 1.77–1.80; *p* < 0.001).

### 3.2 Body composition and blood pressure

Between-group comparisons revealed significant differences in weight and BMI changes, favoring the GFD-G over the MD-G (*p* = 0.018 and *p* = 0.026, respectively). No significant between-group differences were observed for fat mass, fat-free mass, or blood pressure parameters (Table 1).

**Table 1:** Body composition and blood pressure at baseline and following the nutritional concepts.

Parameter	GFD-G (*n* = 6)			MD-G (*n* = 9)			*p* value	*r*_groups_
	T0	T6	*r*_within_	T0	T6	*r*_within_		
Weight [kg]	83.8 [75.8–90.9]	81.0 [74.5–88.3]*	0.791	74.8 [68.8–87.6]	75.5 [68.4–87.2]	0.033	**0** **.** **018**	0.569
BMI [kg·m²]	24.2 [23.0–26.1]	23.4 [22.5–25.4]*	0.796	23.5 [22.1–27.6]	23.7 [21.8–27.5]	0.030	**0** **.** **026**	0.549
Fat free mass [kg]	65.4 [63.4–69.3]	65.3 [63.9–68.6]	0.607	61.8 [57.2–66.6]	64.6 [58.1–67.1]	0.301	0.416	0.204
Fat mass [kg]	16.1 [12.9–21.4]	15.2 [10.6–18.5]*	0.134	13.4 [10.1 −18.1]	12.1 [7.95–17.3]	0.395	0.388	0.306
Fat free mass [%]	80.5 [75.7–84.1]	81.7 [78.5–85.8]*	0.534	81.6 [78.8–86.0]	82.7 [80.2–88.3]*	0.435	0.846	0.096
Fat mass [%]	19.6 [15.9–24.4]	18.4 [14.2–21.5]*	0.534	18.4 [14.0–21.2]	17.3 [11.8–19.8]*	0.435	0.846	0.096
BP sys [mm Hg]	127 [118−135]	128 [123−134]	0.080	127 [119−136]	132 [117−141]	0.133	0.607	0.152
BP dia [mm Hg]	74 [70−88]	73 [70−77]	0.223	80 [74−84]	78 [70–85]	0.093	0.388	0.229

Data represent Median [IQR]. BMI, body mass index; BP sys/dia, systolic/diastolic blood pressure; *r*_within_, effect size for comparison between baseline and final examination within groups; *r*_groups_, effect size for comparison between groups.

*p* value indicates differences between groups with Mann–Whitney *U*-test.

* *p* < 0.05, within the group from baseline to final examination. Bold numbers represent statistical significance of the efficacy endpoints.

Within-group analyses showed that participants in the GFD-G experienced reductions in weight, BMI, and fat mass, along with an increase in fat-free mass percentage. In the MD-G, significant improvements were observed only in fat mass percentage and fat-free mass percentage, while weight and BMI remained unchanged. Systolic and diastolic blood pressure did not change significantly in either group (Table 1).

### 3.3 Dietary assessment

Table 2 summarizes the nutritional patterns of the intervention groups at baseline and during the intervention period. At baseline, there were no statistically significant differences between the groups in mean energy or nutrient intake, except for the intake of saturated fatty acids.

**Table 2:** Dietary patterns of the GFD-G and MD-G at baseline and during intervention.

Dietary Components	GFD-G (*n* = 6)		MD-G (*n* = 9)		*P* value	AR/AI (EFSA)
	Baseline	Intervention	Baseline	Intervention		
Energy, Macronutrients and fibers						
Energy [kcal]	2,013 [1,573–2,416]	2,232 [1,828–2,357]	2,710 [1,830–3,294]	2,500 [1,993–3,081]	0.335	2,338–3,340 (AR)
Carbohydrate [g]	244.2 [170.4–264.3]	210.1 [193.6–254.2]	276.5 [187.8–323.3]	230.8 [186.8–331.5]	0.299	
Carbohydrate [%]	47.1 [39.9–50.8]	43.2 [38.7–48.2]	44.3 [37.6–48.7]	44.3 [37.6–48.7]	0.998	
Fat [g]	69.4 [51.0–104.8]	81.9 [52.7–95.0]	92.7 [74.0–139.3]	93.0 [77.9–125.3]	0.200	
Fat [%]	35.3 [26.1–39.0]	33.3 [25.1–39.1]	36.2 [31.4–42.0]	36.2 [31.4–42.0]	0.284	
Protein [g]	84.4 [73.7–89.5]	92.8 [71.1–149.5]	88.2 [69.8–142.8]	94.6 [77.2 –125.8]	0.627	
Protein [%]	15.5 [14.0–21.8]	17.6 [15.0–26.0]	16.7[14.4–17.4]	16.7 [14.4–17.4]	0.094	
Fiber [g]	21.5 [11.8–29.1]	21.2 [18.0–38.0]	30.0 [15.9–36.2]	25.3 [17.1–34.9]	0.518	25 (AR)
Fatty acids						
SFA [mg]	25.8 [16.2–34.5]^†^	29.4 [17.2–32.8]	42.9 [31.3–52.3]	42.4 [31.0–47.3]	0.056	
MUFA [mg]	32.3 [15.1–47.0]	30.0 [15.6–38.2]	28.1 [24.8–52.6]	29.5 [25.1–44.8]	0.490	
PUFA [mg]	17.3 [7.79–21.1]	14.1 [12.5–21.9]	14.4 [9.76–22.5]	16.9 [11.0–19.0]	0.716	∼5%En (AI)^a^
Minerals						
Sodium chloride [g]	7.69 [5.43–28.1]	5.44 [3.60–7.86]	5.27 [3.85–8.80]	7.49 [4.10–8.66]	0.797	
Potassium [mg]	2,848 [2,293–3,211]	3,227 [3,002–5,936]	3,679 [2,429–4,504]	3,546 [2,677–3,807]	0.251	3,500 (AI)
Calcium [mg]	785.1 [592.3–956.9]	924.6 [565.2–1,279]	771.6 [596.3–1,340]	924.2 [677.9–1,056]	0.811	860 (AR)
Magnesium [mg]	326.7 [271.5–455.3]	401.1 [333.2–640.2]	453.2 [311.9–625.5]	444.4 [324.4–467.8]	0.797	350 (AI)
Zinc [mg]	12.1 [9.5–13.1]	13.1 [9.12–18.4]	11.7 [8.64–19.7]	13.2 [9.87–16.0]	0.680	7.5 (AR)
Iron [mg]	11.4 [11.1–14.2]	13.5 [11.1–32.8]	17.7 [10.1–26.7]	16.2 [10.4–19.4]	0.493	6 (AR)
Iodine [µg]	61.9 [43.8–137.8]	84.5 [73.6–303.2]	90.3 [65.3–131.0]	95.7 [85.3–111.9]	0.699	150 (AI)
Vitamins						
Retinol equiv. [µg]	844.8 [599.0–1,473]	1,544 [1,221–1,725]	1,190 [633.8–2,591]	1,160 [774.7–2,164]	0.847	570 (AR)
Vitamin D [µg]	3.30 [1.60–3.73]	1.97 [1.43–3.53]	3.29 [1.76–5.60]	3.19 [1.79–4.49]	0.442	15 (AI)
Vitamin E [mg]	12.2 [8.47–18.6]	12.8 [10.2–19.5]	17.0 [8.03–21.3]	15.2 [10.1–16.6]	0.865	13 (AI)
Vitamin C [mg]	80.7 [34.3–95.6]	124.3 [92.7–150.3]*	104.5 [59.1–190.5]	107.1 [72.2–131.5]	0.638	90 (AR)
Thiamine [mg]	1.08 [0.760–1.53]	1.53 [0.940–2.28]	1.50 [1.28–1.83]	1.70 [1.29–1.89]	0.847	∼1 (AR)
Riboflavin [mg]	1.20 [1.09–1.52]	1.52 [1.10–3.01]	1.70 [1.24–1.83]	1.55 [1.24–1.95]	0.388	1.3 (AR)
Niacin equiv. [mg]	32.5 [28.7–35.7]	41.4 [31.3–84.3]	40.6 [26.9–58.7]	42.0 [32.7–48.3]	0.270	∼15 (AR)
Vitamin B6 [mg]	1.59 [1.34–1.78]	2.10 [1.68–3.03]	2.20 [1.24–2.81]	2.16 [1.49–2.45]	1.000	1.5(AR)
Folic acid [µg]	243.9 [214.0–351.8]	299.5[207.0–388.8]	360.0 [216.1–457.0]	330.6 [214.7–383.2]	0.898	250 (AR)
Vitamin B12 [µg]	4.21 [2.98–5.13]	6.37 [4.80–7.55]	3.69 [2.77–9.04]	5.03 [3.14–8.44]	0.624	4 (AI)

Data are presented as Median [IQR]. SFA, saturated fatty acids; MUFA, monounsaturated fatty acids; PUFA, polyunsaturated fatty acids.

^a^ Based on EFSA AIs for alpha-linolenic acid, linoleic acid, eicosapentaenoic acid, and docosahexaenoic acid. AR, average requirement; AI, adequate intake; EFSA, European food safety authority.

* *p* < 0.05, within the group from baseline to final examination; *p* value indicates differences between groups.

^†^ *p* < 0.05 between groups at baseline.

When comparing intervention assessments to baseline values, the median energy intake in the GFD-G was approximately 100 kcal higher, whereas it was approximately 100 kcal lower in the MD-G. Compared to baseline, the GFD-G showed a non-significant increase in protein intake at the intervention assessments, along with improved micronutrient intake, including a significant increase in vitamin C consumption.

In the MD-G, no significant changes in dietary patterns were observed.

No statistically significant differences in energy or nutrient intake were observed between the groups at the intervention assessments.

In contrast to baseline assessment, the proportion of participants below the AR in the GFD-G was lower at the intervention assessment for several micronutrients, including fiber (50.0 → 16.7%), calcium (66.7 → 33.3%), retinol equivalent (16.7 → 0%), vitamin C (66.7 → 16.7%), riboflavin (83.3 → 33.3%), vitamin B6 (33.3 → 0%), and folate (50.0 → 33.3%). Energy (50.0%), zinc (0.0%), iron (0.0%), thiamin (33.3%), vitamin E (50.0%) and niacin (0.0%) remained unchanged. In the MD-G, proportions below the AR for calcium (55.6 → 44.4%) and vitamin C (44.4 → 33.3%), vitamin E (33.3 → 22.2%), vitamin B6 (33.3 → 22.2%), were lower, whereas proportions for fiber (22.2 → 33.3%), retinol equivalent (0.0 → 11.1%) and riboflavin (22.2 → 33.3%) increased. Energy (44.4%), zinc (11%), iron (11%), thiamin (11.1%), niacin (11.1%) and folate (22.2%) showed no relevant differences between baseline and the intervention assessment. For nutrients with only an AI, intakes are presented relative to the AI without inferring inadequacy.

### 3.4 Exercise testing

#### 3.4.1 Metabolic outcomes

At baseline, no significant differences were observed between the study groups.

**Figure 3:**
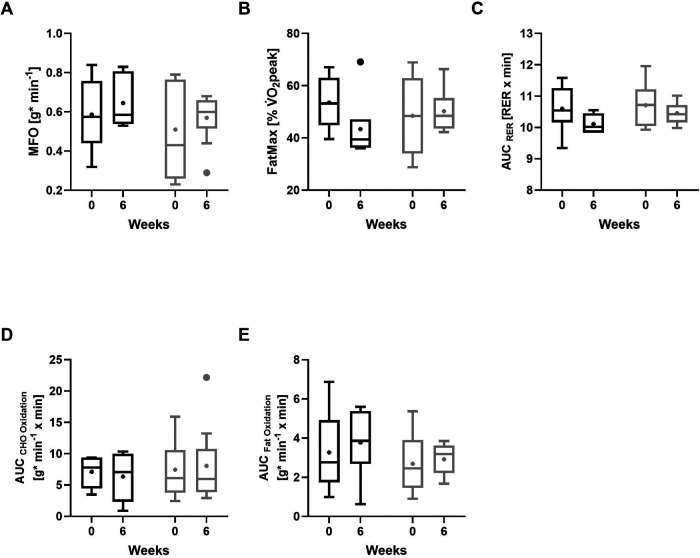
Metabolic outcomes at baseline and after 6 weeks of intervention. **(A)** Maximum fat oxidation (MFO), **(B)** FatMax (exercise intensity at which MFO occurs), **(C)** respiratory exchange ratio (RER; averaged across all completed exercise stages), **(D)** carbohydrate (CHO) oxidation rates up to the individual ventilatory threshold (VT), **(E)** fat oxidation rates up to the individual VT. GFD-G (gluten-free diet group, black); MD-G (Mixed diet group, grey). Data shown as boxplots (Tukey) with median and interquartile range; whiskers represent 1.5 × interquartile range; “+” denotes mean values. Panels **C–E** show values calculated as area under the curve (AUC).

After six weeks of intervention, no significant differences were observed between groups for MFO (Figure 3A; *p* = 0.607; *r* = 0.152) or FatMax (Figure 3B; *p* = 0.181; *r* = 0.365). Similarly, between-group comparisons for the AUC of RER across all stages completed by all participants (Figure 3C; *p* = 0.607; *r* = 0.137), carbohydrate oxidation up to the individual VT (Figure 3D; *p* = 0.776; *r* = 0.076), and fat oxidation up to the individual VT (Figure 3E; *p* = 0.864; *r* = 0.061) did not reach significance.

Within the GFD-G, MFO showed a non-significant change with a medium effect size (Figure 3A; *p* = 0.345; *r* = 0.385), and FatMax showed no statistically significant change, although the effect size was large (Figure 3B; *p* = 0.173; *r* = 0.556). In the MD-G, changes in both MFO (Figure 3A; *p* = 0.374; *r* = 0.296) and FatMax (Figure 3B; *p* = 0.767; *r* = 0.099) were small. The AUC for RER, calculated across all stages completed by all participants, suggested a medium non-significant shift toward lower values in the GFD-G (Figure 3C; *p* = 0.116; *r* = 0.408), whereas no consistent change was observed in the MD-G (*p* = 0.314; *r* = 0.149). When restricting the analysis to intensities up to the individual VT, no significant alterations were observed for carbohydrate oxidation (Figure 3D; GFD: *p* = 0.753; *r* = 0.128; MD: *p* = 0.953; *r* = 0.020) or fat oxidation (Figure 3E; GFD: *p* = 0.345; *r* = 0.385; MD: *p* = 0.515; *r* = 0.217).

#### 3.4.2 Performance-related outcomes

**Figure 4:**
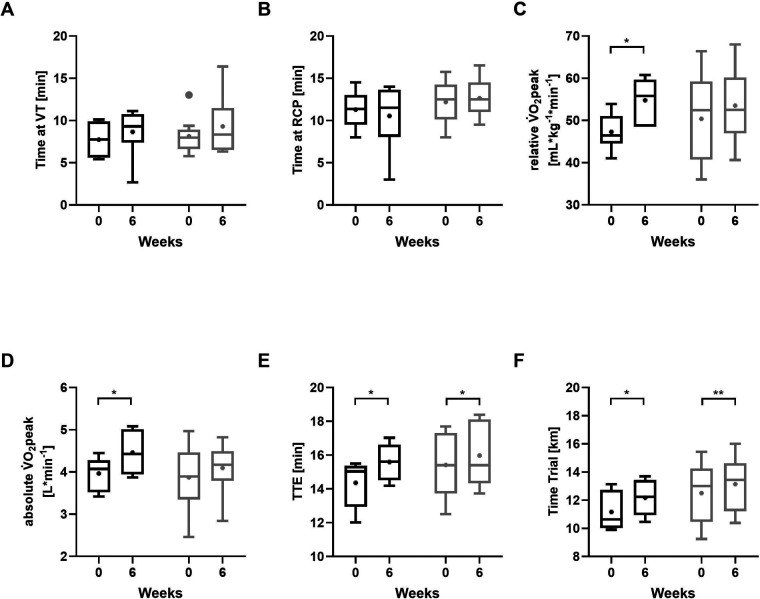
Performance-related outcomes at baseline and after 6 weeks of intervention. **(A)** ventilatory threshold (VT), **(B)** respiratory compensation point (RCP), **(C)** relative peak oxygen uptake (V̇O₂peak), **(D)** absolute peak oxygen uptake (V̇O₂peak), **(E)** time to exhaustion (TTE), **(F)** time trial performance. GFD-G (gluten-free diet group, black); MD-G (Mixed diet group, grey). Data shown as boxplots (Tukey) with median and interquartile range; whiskers represent 1.5 × interquartile range; “+” denotes mean values. *p < 0.05, **p < 0.001, Wilcoxon signed-rank test for differences between baseline and post-intervention (6 weeks).

Except for TTE, none of the performance-related parameters during the ramp incremental exercise test differed notably between groups (all *p* ≥ 0.05; *r* < 0.3), including time at VT (Figure 4A), RCP (Figure 4B), V̇O₂peak (Figure 4C). Evaluation of the data in absolute terms (L/min) indicated a pattern consistent with the relative V̇O₂peak values (Figure 4D): between-group comparison was not significant (*p* = 0.409; *r* = 0.210). TTE (Figure 4E) showed a medium effect favoring the GFD-G (*r* = 0.442) without reaching statistical significance (*p* = 0.088). Between-group differences in improvements during the 60 min time trial (Figure 4F) were medium (*r* = 0.313) but did not reach statistical significance (*p* = 0.328).

Intragroup analyses revealed significant increases in V̇O₂peak in the GFD-G (*p* = 0.046; *r* = 0.640), whereas changes in the MD-G were small (*p* = 0.214; *r* = 0.19). Translating relative V̇O₂peak values to absolute terms (L/min) showed a similar pattern, with a significant increase in the GFD-G (*p* = 0.046; *r* = 0.640) and smaller, non-significant changes in the MD-G (*p* = 0.173; *r* = 0.446). Changes in the time at VT and RCP were small in both the GFD-G and MD-G (all *p* ≥ 0.05; *r* < 0.3). TTE improved significantly in both groups, with large effects in the GFD-G (*p* = 0.046; *r* = 0.651) and MD-G (*p* = 0.015; *r* = 0.610). Distance covered during the 60 min time trial increased significantly in both groups (GFD-G: *p* = 0.027; *r* = 0.620; MD-G: *p* = 0.001; *r* = 0.780).

### 3.5 Gastrointestinal quality of life

Detailed results of the GIQLI subscales and total score are provided in Table 3. No significant baseline differences between the study groups were detected in GIQLI-Scores (*p* = 0.514) Changes in GIQLI -Scores from baseline to post-intervention were small in the GFD-G (*p* = 0.833; *r* = 0.019) and medium in the MD-G (*p* = 0.068; *r* = 0.251). No significant group differences could be detected for changes in the GIQLI -Score (*p* = 0.388; *r* = 0.262).

**Table 3:** GIQLI subscales and total score at baseline and during intervention.

Domain (No. of items)	GFD-G (*n* = 6)		MD-G (*n* = 9)	
	T0	T6	T0	T6
GI symptoms (19)	66.0 [62.5–68.3]	69.5 [67.5–70.8]	65.0 [63.0–68.5]	68.0 [62.5–70.5]
Physical function (7)	28.0 [26.3–28.0]	24.5 [21.0–28.0]	28.0 [28.0–28.0]	28.0 [28.0–28.0]
Emotional well-being (5)	20.0 [18.8–20.0]	17.5 [15.0–20.0]	20.0 [20.0–20.0]	20.0 [20.0–20.0]
Social dimension (4)	16.0 [16.0–16.0]	16.0 [16.0–16.0]	16.0 [16.0–16.0]	16.0 [16.0–16.0]
Therapeutic component (1)	4.0 [4.0–4.0]	4.0 [4.0–4.0]	4.0 [4.0–4.0]	4.0 [4.0–4.0]
Total score (36)	131.5 [128.0–136.0]	130.0 [125.5–138.0]	133.0 [131.0–136.5]	136.0 [130.5–138.5]
*P* value (Wilcoxon)	0.833		0.068	
*P* value (Mann–Whitney)	0.388			

Data are presented as Median [IQR]. For total score: *p* value (Wilcoxon)…within the group from baseline to final examination; *p* value (Mann–Whitney) …Differences between groups with Mann–Whitney *U*-test.

## 4 Discussion

This study was conducted as an exploratory, matched-group pilot trial, designed to provide preliminary evidence and inform the design of future larger studies. Accordingly, statistical analyses were exploratory. Several key principles of the CONSORT extension for pilot trials were applied to ensure transparency and methodological rigor. While previous research predominantly focused on short-term interventions, this study compared the effects of a 6-week GFD and MD, both paired with SIT, on body composition, metabolic markers, and performance outcomes in physically active young men. Significant reductions in weight, BMI, and fat mass (absolute and percentage) were observed in the GFD-G, alongside an increase in fat-free mass percentage. While part of the observed reduction in the GFD group may be attributed to daily fluctuations (about 0.5 ± 0.2 kg) (52), the remaining change—approximately 2%–4% of body weight—falls within the range generally considered clinically meaningful over 6 weeks (53, 54), particularly when accompanied by improvements in body composition. Considering the BIA device's measurement variability (1.3% of body weight, ≈1.1 kg in this cohort), the observed reduction in absolute fat mass (−2.04 kg) exceeded expected error, indicating a true physiological effect.

At baseline, the median weight in the GFD-G was approximately 9 kg higher than in the MD-G, yet reported median energy intake was ∼700 kcal lower. Despite thorough verification and plausibility checks of the dietary records, some underreporting cannot be excluded, which is a known limitation of self-reported data, also in normal-weight athletes (55). The reported intake in the GFD-G was below the EFSA AR for this population (Table 2), creating a discrepancy. This could reflect underreporting or indicate that participants had consumed such low energy intakes only during the short 3-day recording period rather than habitually. In the latter case, the observed energy restriction could plausibly contribute to the reductions in weight and fat mass in the GFD-G.

Whereas the MD-G showed medium reductions in fat mass and a significant increase in fat-free mass percentage, these changes were less pronounced than in the GFD-G, with significant differences between groups for weight and BMI. Studies suggest that SIT can be an effective strategy for improving body composition (56). While a GFD is essential for weight management in individuals with celiac disease, particularly those who are underweight or overweight (57), it does not significantly contribute to weight loss in non-celiac individuals according to the current state of evidence. Although a GFD might lead to a slight reduction in waist circumference and fat percentage, these changes are not substantial enough to suggest a clear benefit (58). Notably, caloric intake during the intervention was slightly higher in the GFD-G (approximately 100 kcal/day more) and slightly lower in the MD-G (approximately 100 kcal/day less), suggesting, based on reported intake, that reductions in weight and BMI in the GFD-G were not solely attributed to caloric restriction during the intervention. Therefore, the more pronounced improvements observed in the GFD-G may be attributed to individual variability in responses or adaptations to the training protocol, rather than to the effects of the diet alone. However, energy expenditure was not directly assessed, which limits the ability to determine whether the observed changes were driven by a negative energy balance.

In the current investigation, systolic and diastolic blood pressure showed no significant changes within or between groups. This is likely due to the participants' baseline normotensive status, as the cohort consisted of healthy, physically active young men with normal blood pressure. While some evidence reports improvements in cardiovascular risk factors with a GFD (59–61), effects on blood pressure appear more pronounced in individuals with pre-existing hypertension or metabolic disturbances (62, 63). Overall, the current findings suggest that a short-term GFD has limited impact on blood pressure in healthy, normotensive adults.

In terms of metabolic outcomes, after six weeks of intervention, no significant differences were observed between groups for MFO or FatMax during the ramp incremental test. Within the GFD-G, MFO showed a non-significant increase with a medium effect size, whereas FatMax tended to decrease slightly, whereas changes in the MD-G were small. Substrate oxidation up to the individual VT showed only subtle changes in the GFD-G and remained largely unchanged in the MD-G, resulting in no significant differences between the groups. The current findings suggest that although maximal fat oxidation may be higher, this peak occurs at a lower treadmill workload (inclination × speed) in the GFD-G. Systematic reviews highlight that methodological factors (e.g., exercise protocol, meal timing, and macronutrient intake) can affect MFO measurement, but do not identify GFD specifically as a variable impacting MFO in healthy individuals (38, 64). Animal studies suggest that GFD may upregulate genes related to fat oxidation and reduce adiposity (65), but these findings have not been translated into evidence for increased MFO during exercise in humans. The lack of consistent changes in the ramp incremental test, coupled with the variability in trends, underscores the need for further investigation in larger cohorts to clarify the metabolic implications of a GFD. Overall, the current results do not provide strong evidence of a conclusive impact on fat metabolism during submaximal exercise.

For performance outcomes, an intriguing finding was that V̇O₂peak improved significantly in the GFD-G, while both groups had significant gains in TTE and the 60 min time trial distance. Translating relative to absolute V̇O₂peak values showed a consistent pattern, indicating that improvements were not solely driven by changes in body weight. Although dietary patterns have been shown to influence V̇O₂peak through modifications in energy availability, macronutrient distribution, micronutrient status, and overall diet quality (66), there is currently no evidence from clinical studies showing the effect of a GFD on V̇O₂peak in non-celiac athletes. One short-term study assessed time trial performance in this population and, similar to our findings, reported no differences between diet conditions (14). Accordingly, in the current investigation, no significant differences between groups were detected in performance-related parameters, suggesting comparable training adaptations despite dietary differences. Performance improvements were primarily attributed to the training intervention. Research supports the hypothesis that SIT positively influences time trial performance, as shown in several studies (32, 67, 68).

Recent studies in non-celiac individuals have investigated the effects of GFDs on athletic performance and metabolic outcomes. Despite the popularity of GFDs among athletes (2), research has not shown any significant improvements in performance, gastrointestinal symptoms, or inflammatory markers in non-celiac athletes following a short-term GFD (14). In line with these findings, no significant differences in GIQLI-Score were observed between the study groups in the current investigation at baseline or in changes from baseline to post-intervention. Changes were small in the GFD-G and medium in the MD-G, but no significant group differences were detected. Given that participants were healthy and reported no baseline gastrointestinal complaints, these findings suggest that the overall impact of the dietary intervention on quality of life may be limited in this context. Despite the commercial hype and athlete testimonials, scientific evidence supporting the nutritional benefits of GFDs in non-celiac athletes remains limited (2). On the contrary, the diet may lack essential nutrients like whole grains and dietary fiber, potentially leading to nutritional deficiencies (69, 70). Participants in the GFD-G generally moved closer to dietary reference values for several nutrients, particularly fiber and vitamin C, with additional improvements in calcium, riboflavin, and folate. However, changes in a single participant substantially influence percentage distribution. Despite improvements, challenges remained in meeting recommendations for iodine, vitamin D, and calcium—nutrients often insufficient in GFD due to the exclusion of certain food groups (10). The MD-G showed only minor improvements for calcium and vitamin C, while fiber and riboflavin intake slightly declined. Overall, the GFD-G displayed a more favorable trend, though confirmation in larger cohorts is needed.

This pilot trial has several limitations that should be considered when interpreting the findings. The small sample size and dropout rate in the GFD-G reduce the power of statistical analyses, although baseline characteristics were well matched and no major post-intervention differences were detected. Outdoor testing may have introduced some environmental variability despite efforts to standardize conditions (same track, similar time of day, calm weather). Additionally, tests conducted at the upper end of the observed temperature range (24–26°C) could have posed an additional challenge for endurance performance; however, this range is generally considered acceptable for field-based running assessments (71). The lack of a non-training control group limits conclusions about diet-specific effects. Although plausibility checks and participant clarifications were conducted, self-reported dietary intake may be prone to underreporting, affecting the accuracy of nutritional data. The absence of mechanistic biomarkers—such as inflammatory cytokines (e.g., IL-6, TNF-α, CRP), gut permeability markers (e.g., zonulin), oxidative stress indicators (e.g., malondialdehyde, glutathione), or gut-related outcomes (e.g., fecal calprotectin, short-chain fatty acids)—is another limitation. Future studies should address these limitations by including mechanistic biomarkers to better understand physiological pathways underlying the effects of gluten exclusion. They should also recruit a larger sample size along with a healthy mixed-diet control group based on nutritional guidelines, as well as non-training participants, to more effectively assess the impact of a GFD on body composition, metabolic health, and performance outcomes in endurance athletes.

## 5 Conclusion

This study examined the effects of a 6-week GFD and MD, combined with SIT, on body composition, metabolism, and performance in active young men. The GFD-G showed significant reductions in weight and BMI compared to the MD-G. Given the higher baseline body weight and generally lower reported energy intake, these changes cannot be attributed with certainty to the dietary intervention itself and are likely related to differences in total energy intake. Metabolic outcomes revealed subtle differences in the GFD-G. However, changes during the intervention were modest and not statistically significant. Performance improvements were similar in both groups, primarily driven by SIT rather than dietary differences.

Nutritional analysis indicated that while the GFD-G improved intake of fiber, and vitamin C, it fell short in meeting key micronutrient needs, such as iodine, vitamin D, and calcium, highlighting potential risks of restrictive diets. The study's limitations, including a small sample size, dropout rates, and absence of a non-training control group, necessitate cautious interpretation of results. Future research with larger cohorts and more robust controls is needed to confirm findings and better isolate the effects of a GFD. Overall, while the GFD showed no clear advantage or disadvantage regarding performance outcomes, its application in non-celiac athletes requires careful consideration due to potential nutritional gaps and limited performance advantages.

## Data Availability

The raw data supporting the conclusions of this article will be made available by the authors, without undue reservation.
